# Evaluating participant experiences of Community Panels to scrutinise policy modelling for health inequalities: the SIPHER Consortium

**DOI:** 10.1186/s40900-023-00521-7

**Published:** 2024-01-08

**Authors:** Ellen Stewart, SIPHER Greater Manchester Community Panel, SIPHER Scotland Community Panel, SIPHER Sheffield Community Panel, Elizabeth Such

**Affiliations:** 1 University of Strathclyde, Glasgow, UK; 2 University of Nottingham, Nottingham, UK

**Keywords:** Data-intensive research, Policy modelling, Public involvement, Evaluation, Health inequalities

## Abstract

Data-intensive research, including policy modelling, poses some distinctive challenges for efforts to mainstream public involvement into health research. There is a need for learning about how to design and deliver involvement for these types of research which are highly technical, and where researchers are at a distance from the people whose lives data depicts. This article describes our experiences involving members of the public in the SIPHER Consortium, a data-intensive policy modelling programme with researchers and policymakers working together over five years to try to address health inequalities. We focus on evaluating people’s experiences as part of Community Panels for SIPHER. Key issues familiar from general public involvement efforts include practical details, careful facilitation of meetings, and payment for participants. We also describe some of the more particular learning around how to communicate technical research to non-academic audiences, in order to enable public scrutiny of research decisions. We conclude that public involvement in policy modelling can be meaningful and enjoyable, but that it needs to be carefully organised, and properly resourced.

## Abstract (plain language summary)

Actively involving members of the public is less common in ‘data-intensive health research’ (health research which does not create new data but focuses on analysing big existing datasets of statistics) than in conventional health research. ‘Computational policy modelling’ is an example of data-intensive health research where public involvement is not yet standard practice. This article describes our experiences involving members of the public in the SIPHER Consortium, a policy modelling programme with researchers and policymakers working together over five years to try to address health inequalities. This paper focuses on evaluating people’s experiences as part of Community Panels for SIPHER. We brought together people with lived experience of health inequalities into three Community Panels, and we met for half a day 3-4 times a year to discuss and give feedback on the research. At first, it was difficult for Panel members to understand the research. Researchers had to try harder to avoid jargon, explain their work in plain English, and focus on the impact of the research in the ‘real world’. Both the researchers and the Panel members learned how to communicate better over repeated meetings. Over time, we managed to have meaningful discussion of the choices researchers were making, so Panels could see their impact on the research. It was important that details of the meetings – planning meetings carefully so everyone feels welcome and valued, providing support with digital technology, financially rewarding people for their time – were taken seriously. We conclude that public involvement in policy modelling can be meaningful and enjoyable, but that it needs to be carefully organised, and takes time and money to get right.

## Background

Policy modelling is an increasingly influential type of evidence for decision-makers in the UK [1]. The SIPHER Consortium is a collaborative research consortium that seeks to develop new modelling tools which will address population health inequalities [2]. SIPHER coproduces these modelling tools by working in partnership with three policy organisations working at different geographical levels in the UK: the Scottish Government, Greater Manchester Combined Authority, and Sheffield City Council [3]. This paper summarises our experiences of involving members of the public in our research, with a focus on Community Panel members’ experiences of the process.

Policy modelling is an example of data-intensive health research, relying on the linkage and analysis of ‘Big Data’ datasets which have been ‘deidentified’; that is, the link between a datapoint, and the human person it represents, has been broken. Public involvement is both particularly important, and particularly challenging in this kind of research [4]. A recent review identified that “mathematical and economic modelling has not embraced the potential of active public involvement, either philosophically, in terms of perceiving any value, or methodologically in practice” [5]. Policy modelling techniques have developed in a set of academic disciplines – for example, computer science and engineering – where directly involving patients and members of the public in research is not yet mainstream. That is, while applied healthcare researchers and medical research are likely to receive significant guidance from their institutions, funders and academic outlets about involving patients and the public [6], modellers are less likely to have this institutional background. There might be an assumption that responsibility for public engagement sits with data collection, rather than with its analysis. The work of modelling is also technical and numerical, which can be off-putting for broader audiences and feel removed from ‘real life’ [7]. There is nonetheless a formal requirement in the UK for modellers to involve wider publics: the UKRI is clear that “Public involvement is important, expected and possible in all types of health and social care research” [8] and the UK Standards for Public Involvement in Research apply to all health and social care research.

In the context of public health, modelling has been criticised for neglecting questions of accountability and transparency [9]. There have been efforts to ‘open up’ what Stewart & Smith [1] described as the ‘black magic’ of policy modelling for health, but these tend to focus on a) consulting members of the public about what interventions should be modelled or b) consulting members of the public about *how to communicate* modelling to broader publics. Each of these, while important, leaves the internal choices and assumptions of the models unexamined by members of the public. In SIPHER, we sought to open up policy modelling research for critical scrutiny by members of the public, especially people with lived experience of socio-economic disadvantage and health inequalities. Given the technical and mathematical nature of much of the modelling process, this aim has particular challenges which we will discuss in this paper. The paper is collaboratively co-authored by Stewart, the co-Investigator who organised the Community Panels, the Community Panel members, and Such, who leads on evaluation of SIPHER.

Our approach to involving members of the public in the research was as follows. Since autumn 2020 we have worked with a community organisation in each of Sheffield, Scotland (Fife) and Greater Manchester. The SIPHER Consortium was launched in late 2019, and so the Panels were not in place when the research funding application was designed, nor for the first months of the consortium. Our community partner organisations have helped us to recruit and support a group of local people to stay involved with SIPHER across the duration of our research programme. No specialist knowledge beyond people’s own lived experience of inequalities in their local area was required for membership. Working in partnership with trusted community organisations has helped us to recruit and sustain Panels including people with experience of recovery from addiction, serious mental health issues, homelessness and unemployment. We meet regularly for workshops to discuss and gain feedback on the research, and workshops were designed to be inclusive in a number of ways. When the COVID-19 pandemic hit, workshops had to be held mostly online, and we were able to provide people with simple tablets and tailored IT support to get to grips with them. Once in person workshops became possible, decisions about meeting in person or continuing online have been informed by Panel member preferences. In line with National Institute for Health Research guidance, all Panel members are offered a modest financial honorarium for taking part in each workshop, as well as travel and/or data expenses being covered. We also pay a fee to the community organisation partner for their efforts in recruiting and providing ongoing support to the Panels. Ethical approval for collecting some data (such as notes of workshops) was awarded by the University of Edinburgh School of Social and Political Sciences Research Ethics Group.

## Main text

This basic model of Community Panels has worked well in practical terms. All of our community partner organisations have continued to support SIPHER and the majority of Panel members who have been involved have stayed involved. Given the project’s challenging duration in the context of a global pandemic (2020–2023), we feel this is a positive outcome. Those who have left – 1 of 8 members in Sheffield, 4 of 9 members in Greater Manchester, and 2 of 10 members in Scotland – have overwhelmingly had to leave due to changes in their own circumstances (especially moving into paid work which cannot accommodate regularly attending meetings, or serious health issues). Only one Panel member left stating dissatisfaction with the experience, and this was in the context of withdrawing from their involvement with the community organisation partner generally. We have recruited some new members across all three Panels, although numbers in our Greater Manchester panel are at the time of writing rather low.

Table 1 shows the topics of the workshops we have held in the last two years. These topics have ranged across the scope of SIPHER’s work, both ‘inside’ and ‘outside’ the models themselves: informing the choice of ‘indicators’ used to measure progress on policy goals; identifying factors missing from systems maps; critiquing demonstrations of ‘decision support tools’ for policymakers. Alongside this, Panels have played more conventional involvement roles such as advising on plain English communication of SIPHER’s work, and piloting survey tools.

**Table 1:** Chronological list of SIPHER Community Panel workshops and topics up to summer 2023

Date	Topic(s)
Autumn 2020	Introductions Connecting work and health: the idea of ‘good work’
Spring 2021	Systems mapping for inclusive economies Choosing indicators for inclusive economies
Autumn 2021	Creating a public-friendly Information Pack for SIPHER
Winter 2021/2	Improving survey questions for Workstream 6 (public views on inequality)
Spring 2022	Decision Support Tool Overall SIPHER consortium update
Autumn 2022	Systems mapping for housing and health
Spring 2023	Cost-of-living responses modelling Mid-point evaluation

## How we evaluated Panels

Informed by SIPHER’s broader mid-point evaluation, we identified a series of accessible questions to ask Panel members to understand their experiences of involvement in the research:

1. What is the first word that pops into your head when you think of SIPHER?
2. What were your hopes for being involved in SIPHER’s Community Panels?
3. What has gone well, and what has gone badly?
4. What would you like SIPHER to do differently going forward?

These questions were shared in Panel workshops in March and April 2023, and Panel members were given time to discuss them without any of the SIPHER researchers in attendance. This let Panels have a collective discussion about how things have gone, and while Panel members may have wished to avoid offending us by sharing very critical feedback, we have worked to cultivate a respectful space for dialogue over the years, and the option of anonymously sharing a collective view did enable criticisms to be shared. Members of the Panel summarised the discussion and sent this to Stewart, and Panel members who weren’t able to make the session were offered the opportunity to answer the questions by email. All the answers received were coded (ie grouped into themes) using Nvivo software, and then a draft version of this paper was shared with panel members and discussed together. In total, 4 members of the GM panel, 8 members of the Sheffield panel, and 8 members of the Scotland panel contributed to the evaluation discussion and have authored this article. This section describes key messages from the evaluation. Some of these echo the findings of established research on public and patient involvement in health research, and others seem more specific to data-intensive policy modelling. We also offer some ‘top tips’ from our shared experience.

## Hoping to make a difference

When asked what people’s hopes for their involvement had been, the biggest group of responses emphasised that people joined SIPHER’s Community Panels because they wanted to make a difference on issues around inequalities they could see in society and locally in their communities:

- “*use lived experience to improve things for the future*”
- “*most interested in improving things for others*”
- “*something exciting to be involved with that may make a difference*”
- “*want to influence policy makers to make the right choices*”

Other answers talked about learning and understanding ‘the system’ better. “*More curious than anything, trying to understand the system better, heard so many stories about people’s difficulties and couldn’t understand why*”. “*I've also learned about social policy and how decisions are made which has been interesting.*” Members added that learning was not only from the research and researchers, but a collective process of learning from other Panel members, too: “*To hear people's lived experience and the variety of ideas and views*”. Sharing one’s own experiences was also a motivation: “*to contribute my story*”; “*to have my voice heard*”.

## Paying attention to the ‘not so little’ things

Our evaluation supported the findings of other studies that practical details of involvement can transform people’s experiences of the process, for better or worse [10]. There were generally positive views on the experience of Panel meetings as a friendly space with a sense of a developing team: “*We are a good team now – diverse, vocal and have opinions”; “Developed and helped each other give a context, different strengths and angles have been helpful*.” Over time, Panels have cohered and become increasingly supportive spaces:

“*How we’ve structured it and taken time. Like [another Panel member] said, we’re a bit like a family now. We’re kind of really familiar with each other and got to know each other.*”

In difficult discussions, as well as the facilitator helping, Panel members could check in with, reassure and support each other, and because many of them knew each other outside of the Panel, this support continued beyond the meetings. Generally, Panel members said they felt valued as part of SIPHER. This was partly because of payment (see below), but also because meetings were friendly and welcoming: “*I really like the way you’ve run the sessions, always listening to everyone and valuing all our contributions*”.

Several members across all Panels stated that the payment for taking part was a part of their motivation for getting and staying involved: both because the money was helpful in itself during a difficult period (first the pandemic and then the ‘cost of living crisis’), and because it showed that Panel members are valued by researchers. In the Scotland panel, the only Panel where for organisational reasons shopping vouchers were offered instead of an honorarium, members raised many issues with the shopping voucher system, especially where depending on a generic voucher provider. While Panel members appreciated a change to offer a choice of voucher from their preferred retailer instead, everyone (researchers and participants) agreed that vouchers are a poor solution. Panel members also said that payment alone, without also creating a comfortable environment for people to take part in, would not be enough: “*you can pay people well, but that only goes so far. You want the person, like you have, to structure it well and take time*.”

There were mixed, and to some extent, incompatible views about the impact of meeting mostly online. Few Panel members were confident with Zoom and similar platforms in 2020, and difficulties getting into online meetings made people feel like they were ‘catching up’ when they finally joined. Some topics and materials were more suitable for an online meeting than others. For example, an early online session on systems mapping of ‘inclusive economies’, based on an online map with hundreds of nodes, proved really difficult for everyone to connect with. Eighteen months later, we ran a second in person session on systems mapping, this time on the topic of housing and health. Both being able to work together round tables with paper and pens, rather than via mapping software, and starting with a blank page and Panels’ own experiences, made the second session much more enjoyable and valuable: “*We were talking about housing, but then, in our particular group, it, it were brought up about people who are Black and the particular challenges they are facing. It was about the challenges what people living in poverty are surrounded by. And if you remember, we had a really complex discussion. I think it helped that we were face to face.*” While both the more applied topic and learning from two years of working together helped, Panel members felt that meeting in person allowed for more interaction, and that this helped everyone to speak up and be heard.

Panel members also acknowledge that in person meetings might be especially hard for people managing health issues, or caring responsibilities. In SIPHER’s Community Panels we have collectively decided to keep some online meetings to facilitate the continued engagement of Panel members who struggle to attend in person. Online meetings also have to be run quite carefully to be inclusive, including more frequent breaks than in an in person context and, for participants with hearing loss, providing professional captioning [11]. These practical details of involvement are widely identified as necessary for best practice, and our evaluation suggests they are of even greater significance where the research being discussed is technical and can seem remote from people’s lived experience.

## Learning together

The SIPHER Consortium’s core work is computational modelling of policies, which is highly technical and often expressed numerically. The use of acronyms and jargon is widespread across the research consortium, and Panel members pointed out that even the name ‘SIPHER’ (an acronym of Systems Science in Public Health and Health Economics Research) sounds opaque and inaccessible. Both researchers and Panel members have learned lots, over time, in order to allow participation in workshops to be as meaningful as possible. This has been a steep learning curve. There was a shared view that early Panel meetings were not always accessible in the language and technical details used. Some of these comments emphasised the language that SIPHER researchers use and assume others will understand: “*it seems we have all struggled to understand the language being used”; “Lots of jargon, can be embarrassing to say I don’t know what they were talking about*”. Multiple members noted an improvement over time in the accessibility of content in the sessions: some of this was Panel members developing a sense of the research, but also the researchers learning to communicate more effectively.

Online meetings made it easier for SIPHER researchers from all over the UK to attend Community Panel workshops. However this ease could lead to a ‘business as usual’ approach, and a risk that overly technical presentations not designed for a public audience were repurposed from one online meeting to another. When Panels meet in person, the setting (usually a community centre or other community space) is noticeably different from other types of meeting a researcher might attend, and travel time from university spaces to community spaces also enforces a degree of separation. This helps underline the different audience (public not academic or policymakers) and purposes of the session (listening and creating dialogue, rather than quickly sharing information and making decisions). Online sessions took careful planning and facilitation to break routines in the more technical and jargon-fuelled way that researchers routinely talk about research between themselves within SIPHER.

Mutual learning required ongoing (not one-off) engagements with the Panels. Longer term relationships supported Panel members to be critical of the process where necessary. Several members mentioned that they had been pleasantly surprised by the facilitator’s response to concerns about Panel meetings being too technical and using language that didn’t include everyone: “*Great response to our initial concerns and learning styles. It’s taken a while but we’re feeling more like we know what’s happening, that we’re going in the right direction.*” One Panel member gave the example of a SIPHER researcher saying ‘does that make it clearer?’ after explaining something technical, and the Panel member feeling able to say ‘no, it doesn’t’: they felt this was a positive dynamic where people could be honest. In discussion, it was also pointed out that language varies everywhere: “*with the jargon, you're not always going to get it right. This, it's made me think we have bread cakes in Sheffield. You know, in Preston they’re called barm cakes! So you’re never gonna get it right all the time. So I think, I think we're doing it quite well*.”

**Fig. 1:**
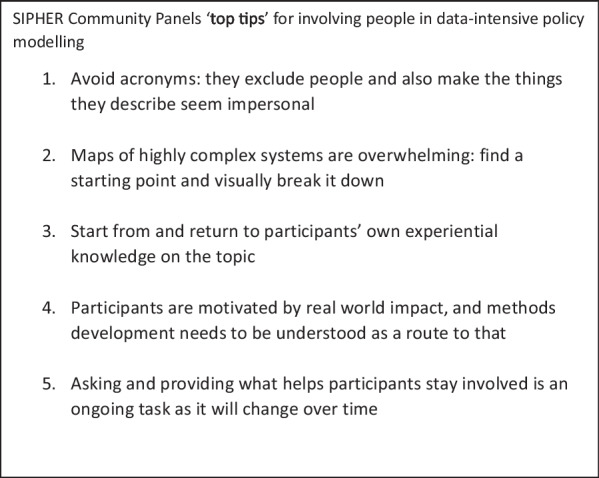
SIPHER Community Panels ‘top tips’ for involving people in data-intensive policy modelling

An alternative perspective from a smaller number of members within the Panels questioned whether the plan for the Panels had been too ambitious, and that even with simpler language, modelling research is too technical and abstract for a non-academic audience to engage with the substantive decisions of the modelling. Most members suggested that more effort to communicate the research overall at the start of the process would have helped, but one stronger view from a Panel member was simply that: “*I don’t think it was necessary to expose participants to the nature of the models/modelling*”. Another Panel member, though, said “*people from my background never gets asked to do things like this, so it’s nice to see inner workings of it*”. As the modelling SIPHER undertakes has matured, it has also become more applicable to practical policy decisions which has helped Panel members to see the potential value of the research. Some members therefore shared a feeling recent sessions were more engaging: both because the research has reached the point of offering results about possible policies (for example, modelling of different policy interventions for the cost of living crisis), and because the Panels understand the process better to engage with it: “*The modelling is now at a point where there are options to explore, and we can see the value* (Fig. 1).”

## Conclusions

In this case study report of involving people in data-intensive policy modelling research, we found that much existing PPI knowledge about how to build relationships with participants remained relevant. Key learning of existing PPI scholarship about relational and practical work [10, 12] is, if anything, more significant in technical data-intensive research that can seem remote from people’s lives. Apparently logistical details of research engagement – when and where to meet, how to organise a meeting, how to compensate people for their time – can seem like administrative work, and problematically seen as therefore not ‘intellectual’ work. This is related to the frequent outsourcing of public and patient involvement work to junior, precariously-employed members of the academic team, and to professional services colleagues who may not be given a central role within the research [6]. Our evaluation showed how far getting practical things right or wrong are fundamental to creating what Staniszewska et al. describe as a ‘deliberative knowledge space’ for these engagements [5]. A difficult entry into a meeting might make the difference between someone who struggles with anxiety feeling able to speak up in a meeting, or not. Requiring a cumbersome system of shopping vouchers might reduce someone’s motivation for taking part, or even worse, make them feel less able and confident in their own lives. In technical, data-intensive research it can be *more* difficult to create welcoming and inclusive spaces, requiring relational skills but also resources of staff time, budget for participant compensation and conducive meetings whether online or in person.

Engaging members of the public with the substance of modelling is not the only option available to projects like SIPHER: the MEMVIE Framework suggests five potential areas of public contribution in modelling projects [5]. Panels could have met only to discuss how to communicate the research to non-academic audiences. One 2014 study found this option the most popular among biomedical researchers, who were resistant to ceding substantial power over research decisions [13]. A more empowered role than SIPHER offered would have included Panels being involved earlier to have input into what topics SIPHER would choose to model: “*I hoped that we would have more input into what is being studied rather than studying what has already been decided upon to study.”* SIPHER’s policy topics were defined in collaboration with our policy partner organisations early in the funding application process [3], before Community Panels were established, and therefore they had no opportunity for input into these decisions. However, given the technical research that SIPHER undertakes and the unfamiliarity of many SIPHER colleagues with public involvement in research, Panel members feel we have made good headway in both understanding and shaping the research. This has required a model of long-term, ongoing engagements and mutual learning, which could not have been achieved through one off consultative dialogues.

## Data Availability

Not applicable.
